# Presentation outside office hours does not negatively influence treatment times for reperfusion therapy for acute ischemic stroke

**DOI:** 10.1007/s00415-020-10106-z

**Published:** 2020-07-31

**Authors:** A. E. Groot, H. de Bruin, T. T. M. Nguyen, M. Kappelhof, F. de Beer, M. C. Visser, C. P. Zwetsloot, P. H. A. Halkes, J. de Kruijk, W. D. M. van der Meulen, T. C. van der Ree, V. I. H. Kwa, S. M. van Schaik, L. Hani, R. van den Berg, M. E. S. Sprengers, S. D. Roosendaal, B. J. Emmer, P. J. Nederkoorn, C. B. L. M. Majoie, Y. B. W. E. M. Roos, J. M. Coutinho

**Affiliations:** 1 Neurology, Amsterdam University Medical Center, University of Amsterdam, Meibergdreef 9, 1105 AZ Amsterdam, The Netherlands; 2 Neurology, Leiden University Medical Center, Albinusdreef 2, 2333 ZA Leiden, The Netherlands; 3 Radiology and Nuclear Medicine, Amsterdam University Medical Center, University of Amsterdam, Meibergdreef 9, 1105 AZ Amsterdam, The Netherlands; 4 Neurology, Spaarne Gasthuis, Boerhaavelaan 22, 2035 RC Haarlem, The Netherlands; 5 Neurology, Amsterdam University Medical Center, Vrije Universiteit Amsterdam, Boelelaan 1117-1118, 1081 HV Amsterdam, The Netherlands; 6 Neurology, Dijklander, Waterlandlaan 250, 1441 RN Purmerend, The Netherlands; 7 Neurology, Noord-West Ziekenhuisgroep, Wilhelminalaan 12, 1815 JD Alkmaar, The Netherlands; 8 Neurology, Tergooi, Van Linschotenlaan 35, 1212 DR Hilversum, The Netherlands; 9 Neurology, Rode Kruis, Vondellaan 13, 1942 LE Beverwijk, The Netherlands; 10 Neurology, Dijklander, Maelsonstraat 3, 1624 NP Hoorn, The Netherlands; 11 Neurology, OLVG-Oost, Oosterpark 9, 1091 AC Amsterdam, The Netherlands; 12 Neurology, OLVG-West, Jan Tooropstraat 164, 1061 AE Amsterdam, The Netherlands; 13 Neurology, Noord-West Ziekenhuisgroep, Huisduinerweg 3, 1782 GZ Den Helder, The Netherlands

**Keywords:** Acute ischemic stroke, Off-hour presentation, Functional outcome, Treatment times, Workflow

## Abstract

**Background:**

Treatment outside office hours has been associated with increased workflow times for intravenous thrombolysis (IVT) in acute ischemic stroke (AIS). Limited data suggest that this “off-hours effect” also exists for endovascular treatment (EVT). We investigated this phenomenon in a well-organized acute stroke care region in the Netherlands.

**Methods:**

Retrospective, observational cohort study of consecutive patients with AIS who received reperfusion therapy in the Greater Amsterdam Area, consisting of 14 primary stroke centers and 1 comprehensive stroke center (IVT: 2009–2015, EVT: 2014–2017). Office hours were defined as presentation during weekdays between 8 AM and 5 PM, excluding National Festive days. Primary outcome was door-to-treatment time (door-to-needle [DNT] for IVT, door-to-groin [DGT] for EVT). For DGT, we used the door time of the first hospital. Other outcomes were in-hospital mortality, modified Rankin Scale (mRS) score at 90 days and symptomatic intracranial hemorrhage (sICH). We performed multivariable linear and logistic regression analyses and used multiple imputation to account for missing values.

**Results:**

In total, 59% (2450/4161) and 61% (239/395) of patients treated with IVT and EVT, respectively, presented outside office hours. Median DNT was minimally longer outside office hours (32 vs. 30 min, *p* = 0.024, adjusted difference 2.5 min, 95% CI 0.7–4.2). Presentation outside office hours was not associated with a longer DGT (median 130 min for both groups, adjusted difference 7.0 min, 95% CI − 4.2 to 18.1). Clinical outcome and sICH rate also did not differ.

**Conclusion:**

Presentation outside office hours did not lead to clinically relevant treatment delays for reperfusion therapy in patients with AIS.

**Electronic supplementary material:**

The online version of this article (10.1007/s00415-020-10106-z) contains supplementary material, which is available to authorized users.

## Introduction

The beneficial effect of intravenous thrombolysis (IVT) and endovascular treatment (EVT) in patients with acute ischemic stroke (AIS) is highly time dependent and a reduction in treatment times increases the chance of good clinical outcome [1,2]. Stroke patients often present outside of office hours, which may cause treatment delay, for example due to lower staff attendance, and decreased availability of imaging [3]. Previous studies on the existence of such an “off-hours effect” have yielded conflicting results, observing both longer and shorter treatment times for patients presenting outside office hours [4–7]. Moreover, these studies mostly focused on door-to-needle times (DNT) for IVT, and there are limited data available on door-to-groin times (DGT) for EVT [8–10].

The Greater Amsterdam Area is a densely populated part of the Netherlands with generally well-organized stroke care and short treatment times for reperfusion therapy [11,12]. As a result, we hypothesized that presentation outside office hours would not lead to treatment delays for both IVT and EVT.

## Methods

### Study design

We performed an observational cohort study in the Greater Amsterdam Area, which consists of 13 primary stroke centers (PSC), 1 general hospital, and one comprehensive stroke center (CSC), with a surface area of 2670 km^2^, and 2.76 million inhabitants. The participating hospitals are listed in Supplemental Table 1. All patients with AIS who received reperfusion therapy between January 2009 and December 2015 (IVT) and between April 2014 and December 2017 (EVT) were included.

### Patient selection

Patients were identified from local prospective stroke registries. According to time of arrival at the Emergency Department (ED), we categorized patients as presenting during “office hours” or “outside office hours”. Office hours were defined as Monday until Friday from 8 AM until 5 PM, with the exception of public holidays. If arrival time at ED was not recorded, we estimated the time by subtracting the median door-to-CT time from the time of the non-contrast CT. For patients receiving EVT, categorization was based on the time of presentation at the ED of the first hospital (i.e. for transfer patients the door time of the PSC was used). Because transfer patients have two door times (both door of PSC and CSC), we also categorized these patients based on the time of presentation at the ED of the CSC in a subgroup analysis. Based on the annual number of IVT procedures, hospitals were categorized as low-volume (≤ 24 IVT treated patients per year), medium-volume (25–49) or high-volume (≥ 50), as done previously [13]. Patients with an in-hospital stroke or for whom the time of presentation could not be ascertained were excluded.

### Data collection and outcomes

We collected individual patient data extracted from medical records [12]. The institutional review board of the Amsterdam University Medical Centers, location AMC approved the study and waived the need for written informed consent from individual patients. Primary outcomes were the door-to-needle time (DNT) for patients receiving IVT and door-to-groin time (DGT) for patients receiving EVT. DGT was defined as the time interval between presentation at the first hospital and time of puncture of the groin. For patients that were transferred from a PSC towards the CSC to receive EVT, we used the door time of the PSC. Other outcomes were the modified Rankin Scale scores (mRS) after 3 months, in-hospital mortality, symptomatic intracranial hemorrhage (sICH, according to ECASS III criteria), infections (pneumonia and urinary tract), and Intensive Care Unit (ICU) admission.

### Statistical analysis

We compared patients presenting outside office hours vs. during office hours. The results for IVT and EVT are reported separately and patients who received both IVT and EVT were analyzed in both groups. Baseline characteristics were compared using Chi-square test for categorical variables, independent samples *T* test for normally distributed continuous variables, and Mann–Whitney *U* test for non-normally distributed continuous variables. Time intervals are expressed as medians with interquartile ranges (IQR). For regression analyses, missing data were imputed using multiple imputations by chained equations (MICE) based on relevant covariates and outcome.

We used multivariable linear regression analysis to evaluate the association between presentation outside office hours and door-to-treatment times. Presentation during office hours was used as the reference category. For DNT, we adjusted for the following pre-specified prognostic factors: age, sex, prior ischemic stroke/TIA, use of antithrombotic therapy, pre-stroke mRS, baseline NIHSS, systolic blood pressure, onset-to-door time, and hospital volume. For DGT, we used the same co-variables and additionally adjusted for treatment with IVT. We used multivariable binary logistic regression analysis to evaluate the association between presentation outside office hours and clinical and radiological outcomes, adjusting for the following pre-specified variables: age, sex, prior ischemic stroke/TIA, use of antithrombotic therapy, pre-stroke mRS, baseline NIHSS, onset-to-needle time, hospital volume, and treatment with IVT (for EVT patients only). Finally, we performed a secondary analysis of patients receiving EVT based on the time of presentation at the ED of the CSC. SPSS version 25 was used for all statistical analyses.

## Results

**Fig. 1:**
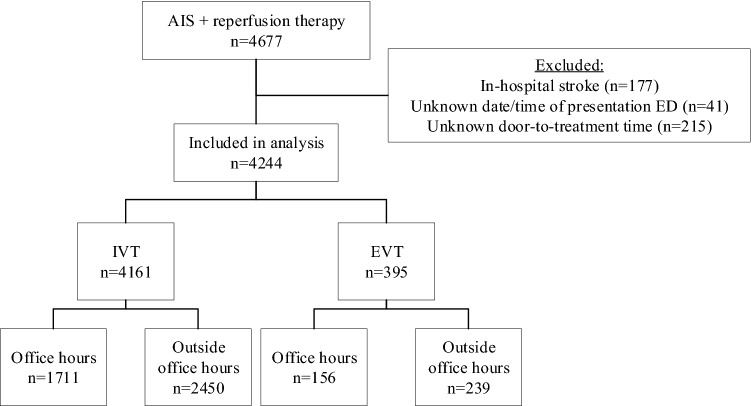
Flowchart for patient selection. There were 311 patients that received both IVT and EVT

In total, 4677 patients with AIS were treated with reperfusion therapy in the study period (Fig. 1). Of these, 433 were excluded because of in-hospital stroke (*n* = 177), unknown time of arrival (*n* = 41), or unknown door-to-treatment time (*n* = 215). Therefore, data of 4244 patients were included in the analysis, of whom 4161 (98%) received IVT and 395 (9.3%) EVT. There were 311/395 (78.7%) patients that received both IVT and EVT. In total, 2450/4161 (58.9%) and 239/395 (60.5%) patients treated with IVT and EVT, respectively, presented outside office hours. The number of EVT patients presented outside office hours increased per year (2014: 38.7%, 2015: 59.2%, 2016: 62.1%, 2017: 64.5%).

Intravenous thrombolysis (IVT) patients who presented outside office hours were more often men (55.5% vs. 51.5%, *p* = 0.010) and were slightly younger (70 vs. 71 years, *p* = 0.015), compared to patients who presented during office hours (Table 1). For patients who received EVT, median onset-to-door time was longer for patients presented outside office hours (62 vs. 51 min, *p* = 0.001). There were no other statistically significant differences between the two groups.

**Table 1:** Baseline characteristics

	Intravenous thrombolysis			Endovascular treatment		
	Office hours, *n* = 1711	Outside office hours, *n* = 2450	*p* value	Office hours, *n* = 156	Outside office hours, *n* = 239	*p* value
Male sex—*n* (%)	880/1709 (51.5)	1358/2446 (55.5)	0.010	88/156 (56.4)	123/239 (51.5)	0.335
Mean age in years ± SD	71 ± 14	70 ± 14	0.015	68 ± 14	67 ± 14	0.841
Mean systolic blood pressure in mmHg ± SD^a^	156 ± 26	155 ± 25	0.178	148 ± 25	145 ± 23	0.232
Mean diastolic blood pressure in mmHg ± SD^b^	85 ± 16	85 ± 16	0.560	83 ± 17	82 ± 14	0.516
Median NIHSS (IQR)^d^	6 (4–12)	7 (4–13)	0.093	17 (13–21)	17 (13–21)	0.886
Pre-stroke mRS ≥ 2—*n* (%)	96/897 (10.7)	121/1301 (9.3)	0.279	8/144 (5.6)	14/210 (6.7)	0.671
Transfer from primary stroke center—*n* (%)	N/A	N/A	N/A	104/156 (66.7)	162/239 (67.8)	0.817
Risk factors—*n* (%)						
Atrial fibrillation	155/1690 (9.2)	221/2403 (9.2)	0.978	28/146 (19.2)	47/212 (22.2)	0.494
Diabetes mellitus	271/1693 (16.0)	383/2402 (15.9)	0.957	25/146 (17.1)	30/212 (14.2)	0.443
Hypertension	729/1688 (43.2)	1016/2401 (42.3)	0.579	64/141 (45.4)	90/209 (43.1)	0.667
Prior ischemic stroke/TIA	448/1691 (26.5)	609/2399 (25.4)	0.426	27/146 (18.5)	28/212 (13.2)	0.173
Medication use—*n* (%)						
Anticoagulation*	37/968 (3.8)	38/1309 (2.9)	0.224	26/144 (18.1)	43/208 (20.7)	0.543
Process measures						
General anesthesia—*n* (%)	N/A	N/A	N/A	43/146 (48.3)	46/231 (51.7)	0.094
Median admission duration in days (IQR)^c^	4 (2–8)	4 (2–8)	0.958	2 (1–5)	2 (1–5)	0.680
Median onset to door (first hospital) time in minutes (IQR)^e^	70 (45–115)	67 (45–112)	0.544	51 (28–74)	62 (44–102)	0.001

*SD* standard deviation, *NIHSS* National Institutes of Health Stroke Scale, *mRS* modified Rankin Scale

^*^Direct oral anticoagulation, vitamin K antagonist or heparin

Number of missing values: ^a^IVT: 91 (5.3%) vs. 136 (5.6%), EVT: 8 (5.1%) vs. 26 (10.9%), ^b^IVT: 91 (5.3%) vs. 140 (5.7%), EVT: 8 (5.1%) vs. 28 (11.7%), ^c^IVT: 29 (1.7%) vs. 52 (2.1%), EVT: 9(5.8%) vs. 26 (10.9%), ^d^IVT: 30 (1.8%) vs. 60 (2.4%), EVT: 10 (6.4%) vs. 28 (11.7%), ^e^IVT: 159 (9.3%) vs. 238 (9.7%), EVT: 7 (4.5%) vs. 7 (2.9%)

### Outcomes IVT

The median unadjusted DNT was slightly longer for patients presented outside office hours than for those who arrived during office hours (32 vs. 30 min, *p* = 0.024, Table 2) and this difference persisted after adjustment for potential confounders (adjusted difference 2.5 min, 95% CI 0.7–4.2). There were no differences regarding in-hospital mortality (7.4% vs. 7.8%, aOR 0.95, 95% CI 0.73–1.23), mRS 0–2 at 3 months (37.8% vs. 36.2%, aOR 1.02, 95% CI 0.87–1.20), and frequency of sICH (4.2% vs. 4.8%, aOR 0.86, 95% CI 0.63–1.18). Patients presenting outside office hours did more often develop pneumonia (7.2% vs. 5.3%, aOR 1.51, 95% CI 1.13–2.00).

**Table 2:** Outcomes for patients treated with intravenous thrombolysis

	Office hours, *n* = 1711	Outside office hours, *N* = 2450	Unadjusted Beta/OR (95% CI)	Adjusted Beta*/OR**, (95% CI)
Median DNT (IQR) in minutes	30 (22–44)	32 (23–45)	1.6 (− 0.2 to 3.4)	2.5 (0.7–4.2)
DNT < 60 min—*n* (%)	1490/1711 (87.1)	2090/2450 (85.3)	0.86 (0.72 to 1.03)	0.78 (0.64–0.95)
DNT < 30 min—*n* (%)	778/1711 (45.5)	1061/2450 (43.3)	0.92 (0.81 to 1.04)	0.86 (0.75–0.98)
Median ONT (IQR)^a^ in minutes	107 (78–160)	108 (80–160)	2.5 (− 1.9 to 6.8)	2.5 (0.7–4.3)
In-hospital mortality—*n* (%)	133/1702 (7.8)	179/2435 (7.4)	0.94 (0.74 to 1.18)	0.95 (0.73–1.23)
Mortality after 3 months—*n* (%)	175/1222 (14.3)	256/1761 (14.5)	1.02 (0.83 to 1.25)	1.10 (0.86–1.42)
mRS 0–2 after 3 months—*n* (%)	519/1432 (36.2)	761/2014 (37.8)	1.07 (0.93 to 1.23)	1.02 (0.87–1.20)
Symptomatic ICH—*n* (%)	80/1661 (4.8)	100/2386 (4.2)	0.87 (0.64 to 1.17)	0.86 (0.63–1.18)
Pneumonia—*n* (%)	90/1711 (5.3)	177/2450 (7.2)	1.40 (1.08 to 1.82)	1.51 (1.13–2.00)
Urinary tract infection—*n* (%)	98/1711 (5.7)	122/2450 (5.0)	0.86 (0.66 to 1.13)	0.90 (0.67–1.20)
ICU admission—*n* (%)	65/1711 (3.8)	102/2450 (4.2)	1.10 (0.80 to 1.51)	1.10 (0.78–1.55)

*DNT* door-to-needle time, *ONT* onset-to-needle time, *OTD* onset-to-door time, *ICH* intracranial hemorrhage, *ICU* intensive care unit

^*^Adjusted for age, sex, prior ischemic stroke/TIA, use of antithrombotic therapy, pre-stroke mRS, baseline NIHSS score, systolic blood pressure, onset-to-door time, hospital volume (for OTD as dependent variable excluding onset-to-door time)

**Adjusted for age, sex, prior ischemic stroke/TIA, use of antithrombotic therapy, pre-stroke mRS, baseline NIHSS, onset-to-needle time, and hospital volume

Number of missing values: ^a^148 (8.6%) vs. 221 (9.0%)

### Outcomes EVT

There was no difference in DGT between patients presenting outside versus during office hours (both 130 min, adjusted time difference 7.0 min, 95% CI − 4.2 to 18.1, Table 3). We also found no difference in DGT for transferred (144 vs. 147 min, adjusted time difference 7.9, 95% CI − 5.2 to 21.1) or directly (103 vs. 89 min, adjusted time difference 5.6, 95% CI − 12.7 to 23.8) presented patients. In-hospital mortality (outside office hours vs. during office hours: 11.7% vs. 16.0%, aOR 1.02, 95% CI 0.47–2.24), mRS 0–2 at 3 months (38.8% vs. 39.7%, aOR 0.92, 95% CI 0.52–1.65), and sICH (9.2% vs. 12.7%, aOR 0.69, 95% CI 0.30–1.57) also did not differ between groups. Fourteen patients arrived at the CSC outside office hours, while initial presentation at PSC was during office hours. In addition, seven patients arrived at the CSC during office hours, while initial presentation at PSC was outside office hours. If we used the door time of the CSC instead of the PSC to categorize patients, there were also no statistically significant differences between the two groups regarding any of the outcomes (Supplemental Table 2).

**Table 3:** Outcomes for patients treated with endovascular treatment

	Office hours, *n* = 156	Outside office hours, *N* = 239	Unadjusted Beta/OR (95% CI)	Adjusted Beta*/OR**, (95% CI)
Median DGT (IQR) in minutes	130 (96–170)	130 (107–175)	6.2 (− 5.2 to 17.6)	7.0 (− 4.2 to 18.1)
Direct patients (*n* = 129)	89 (65–117)	103 (73–122)	6.7 (− 11.1 to 24.5)	5.6 (− 12.7 to 23.8)
Transfer patients (*n* = 266)	147 (119–180)	144 (120–187)	5.1 (− 7.5 to 17.7)	7.9 (− 5.2 to 21.1)
Median DGT (door CSC) in minutes, only transfer patients (IQR)	35 (25–51)	34 (23–52)	− 2.1 (− 11.8 to 7.7)	2.0 (− 8.0 to 12.0)
Median door-to-door time in minutes, only transfer patients (IQR)	102 (79–134)	105 (83–133)	4.1 (− 7.7 to 15.8)	5.6 (− 6.4 to 17.7)
In-hospital mortality—*n* (%)	25/156 (16.0)	28/239 (11.7)	0.70 (0.39 to 1.24)	1.02 (0.47 to 2.24)
Mortality after 3 months—*n* (%)	39/134 (29.1)	61/207 (29.5)	1.02 (0.63 to 1.64)	1.09 (0.55 to 2.16)
mRS 0–2 after 3 months—*n* (%)	58/146 (39.7)	85/219 (38.8)	0.96 (0.63 to 1.48)	0.92 (0.52 to 1.65)
Direct patients (*n* = 129)	24/48 (50.0)	31/70 (44.3)	0.80 (0.38 to 1.66)	0.70 (0.23 to 2.11)
Transfer patients (*n* = 266)	34/98 (34.7)	54/149 (36.2)	1.07 (0.63 to 1.82)	1.01 (0.48 to 2.13)
Symptomatic ICH—*n* (%)	16/126 (12.7)	17/184 (9.2)	0.70 (0.34 to 1.44)	0.69 (0.30 to 1.57)
Pneumonia—*n* (%)	17/156 (10.9)	25/239 (10.5)	0.96 (0.50 to 1.83)	1.77 (0.73 to 4.33)
Urinary tract infection—*n* (%)	5/156 (3.2)	9/239 (3.8)	1.18 (0.39 to 3.59)	1.60 (0.46 to 5.53)
ICU admission—*n* (%)	39/156 (25.0)	38/239 (15.9)	0.57 (0.34 to 0.94)	0.68 (0.35 to 1.33)

*DGT* door-to-groin time, *IQR* interquartile range, *SD* standard deviation, *ICH* intracranial hemorrhage, *ICU* intensive care unit

^*^Adjusted for age, sex, prior ischemic stroke/TIA, use of antithrombotic therapy, pre-stroke mRS, baseline NIHSS score, systolic blood pressure, onset-to-needle time

^**^Adjusted for age, sex, IVT treatment, prior ischemic stroke/TIA, use of antithrombotic therapy, pre-stroke mRS, baseline NIHSS, onset-to-needle time

## Discussion

In this study, 60% of the patients treated with reperfusion therapy presented outside office hours. Presentation outside office hours was associated with a 2 min delay in DNT, which is unlikely to be clinically relevant. Presentation outside office hours was not associated with increased treatment times for patients undergoing EVT. There was also no difference in functional outcome or risk of sICH between patients presenting during versus outside office hours. This emphasizes our hypothesis that in a well-organized acute stroke care region, presentation outside office hours does not necessarily lead to worse outcomes.

A number of studies previously evaluated a possible “off hours” effect in patients receiving IVT [7,14–16]. The results of our study are generally in line with the results from a large European, multicenter cohort (TRISP), which also found a 2 min increase in DNT for presentation outside office hours. Though the increased DNT observed in TRISP was statistically significant, this study also found no association with clinical outcome, providing further proof that these small DNT differences are unlikely to be clinically relevant [7]. While various studies have examined the off-hours effect for IVT, data on this topic for EVT are scarce. Previous studies did report prolonged treatment times for patients with EVT presenting outside office hours, but none of these found an effect on functional outcome at 90 days [8–10,17]. One of those studies used data of the MRCLEAN Registry, reporting on results of all patients treated with EVT between March 2014 and June 2016 in the Netherlands, also including patients from the Greater Amsterdam Area [10]. We, however, report results of patients that received EVT over a wider time span (March 2014 through January 2018), and in contrast, we found no differences in treatment times or functional outcome, regardless of presentation time. Our region may differ from other regions in the Netherlands. Since 2016, all hospitals in our region are committed to a protocol containing requirements for each participating center to ensure optimal 24/7-stroke service (StrokeNet). Implementation of such a protocol may have helped to ensure the timely start of EVT.

Patients that received IVT outside office hours more often suffered from pneumonia. There is at least one other study that also reported this observation [3]. Dysphagia is a common symptom after stroke, and protocols include dysphagia screening to reduce the frequency of aspiration and pneumonia nowadays [18]. While dysphagia screening is routinely done in accordance with national guidelines, such a screening might be delayed in cases who present outside office hours, due to unavailability of specialized staff such as speech therapists.

One of the strengths of our study was that we included all consecutive AIS patients in a large region in the Netherlands, with 15 participating hospitals including both PSCs and a CSC. The in-hospital logistics of these 15 hospitals were comparable, due to the earlier mentioned StrokeNet protocol. Another strength is that we had little missing data for door-to-treatment times. Several limitations of this study should be noted. First, while patients were derived from local prospective stroke registries, a substantial proportion of the data were collected retrospectively. This explains the relatively high proportion of missing data on for example pre-stroke mRS. More importantly, mRS scores at 90 days were missing for 52% of patients treated with IVT, and only for 7.5% of patients treated with EVT. We tried to account for these missing values using multiple imputation. However, the results of functional outcome in IVT patients should be interpreted with caution, as these could be influenced by bias. Third, some patients, especially those transferred from a PSC to a CSC for EVT, initially presented during office hours, but underwent EVT outside office hours. However, the results of the secondary analysis based on the presentation time at the ED of the CSC do not seem to suggest that this led to a substantial distortion of the results. Fourth, even though data on door-to-treatment times were almost complete, we did not collect extra time points such as door-to-imaging times, and therefore, we cannot say anything about other delays that can appear outside office hours. Finally, we did not have data on patients that encountered significant transfer delays and as a result were ineligible for reperfusion therapy. Theoretically, this could differ between patients who presented during vs. outside office hours.

In conclusion, the results of our study suggest that in a well-organized acute stroke care region with overall low DNT and DGT, presentation outside office hours does not necessarily lead to a clinically relevant increase in treatment times for either IVT or EVT.

## Electronic supplementary material

Below is the link to the electronic supplementary material.

Supplementary file1 (DOCX 21 kb)

## References

[1] Saver JL, Fonarow GC, Smith EE, Reeves MJ, Grau-sepulveda MV, Hernandez AF (2013). Time to treatment with intravenous tissue plasminogen activator and outcome from acute ischemic stroke. JAMA.

[2] Fransen PSS, Berkhemer OA, Lingsma HF, Beumer D, van den Berg LA, Yoo AJ (2015). Time to reperfusion and treatment effect for acute ischemic stroke: a randomized clinical trial. JAMA Neurol..

[3] Reeves MJ, Smith E, Fonarow G, Hernandez A, Pan W, Schwamm LH (2009). Off-hour admission and in-hospital stroke case fatality in the get with the guidelines-stroke program. Stroke.

[4] Campbell JTP, Bray BD, Hoffman AM, Kavanagh SJ, Rudd AG, Tyrrell PJ (2014). The effect of out of hours presentation with acute stroke on processes of care and outcomes: analysis of data from the Stroke Improvement National Audit Programme (SINAP). PLoS ONE.

[5] Albright KC, Savitz SI, Raman R, Martin-Schild S, Broderick J, Ernstrom K (2012). Comprehensive stroke centers and the “Weekend Effect”: the SPOTRIAS experience on behalf of the SPOTRIAS investigators. Cerebrovasc Dis.

[6] Jauss M, Schütz HJ, Tanislav C, Misselwitz B, Rosenow F (2010). Effect of daytime, weekday and year of admission on outcome in acute ischaemic stroke patients treated with thrombolytic therapy. Eur J Neurol.

[7] Zonneveld TP, Curtze S, Zinkstok SM, Gensicke H, Moulin S, Scheitz JF, Seiffge DJ, Hametner C, Heldner MR, Traenka C, Erdur H, Baharoglu I, Martinez-Majander N, Pezzini A, Zini A, Padjen V, Correia PN, Strbian D, Michel P, Béjot Y, Arnold M, Leys D, Ringl NPT collaborators (2017). Non-office-hours admission affects intravenous thrombolysis treatment times and clinical outcome. J Neurol Neurosurg Psychiatry..

[8] Mpotsaris A, Kowoll A, Weber W, Kabbasch C, Weber A, Behme D (2015). Endovascular stroke therapy at nighttime and on weekends-as fast and effective as during normal business hours?. J Vasc Interv Neurol.

[9] Almekhlafi MA, Hockley A, Desai JA, Nambiar V, Mishra S, Volny O (2014). Overcoming the evening/weekend effects on time delays and outcomes of endovascular stroke therapy: The Calgary Stroke Program experience. J Neurointerv Surg.

[10] Hinsenveld WH, de Ridder IR, van Oostenbrugge RJ, Vos JA, Groot AE, Coutinho JM (2019). Workflow intervals of endovascular acute stroke therapy during on- versus off-hours. Stroke.

[11] Jansen IGH, Mulder MJHL, Goldhoorn R-JB (2018). Endovascular treatment for acute ischaemic stroke in routine clinical practice: prospective, observational cohort study (MR CLEAN Registry). BMJ.

[12] Groot AE, van Schaik IN, Visser MC, Nederkoorn PJ, Limburg M, Aramideh M (2016). Association between i.v. thrombolysis volume and door-to-needle times in acute ischemic stroke. J Neurol..

[13] Bray BD, Campbell J, Geoffrey CC, Hoffman A, Tyrrell PJ, Wolfe CD (2013). Bigger, faster?: associations between hospital thrombolysis volume and speed of thrombolysis administration in acute ischemic stroke. Stroke.

[14] Fang K, Churilov L, Weir L, Dong Q, Davis S, Yan B (2014). Thrombolysis for acute ischemic stroke: do patients treated out of hours have a worse outcome?. J Stroke Cerebrovasc Dis.

[15] Bodenant M, Leys D, Debette S, Cordonnier C, Dumont F, Hénon H (2010). Intravenous thrombolysis for acute cerebral ischaemia: comparison of outcomes between patients treated at working versus nonworking hours. Cerebrovasc Dis.

[16] Kim SK, Lee SY, Bae HJ, Lee YS, Kim SY, Kang MJ (2009). Pre-hospital notification reduced the door-to-needle time for iv t-PA in acute ischaemic stroke. Eur J Neurol.

[17] Nikoubashman O, Schürmann K, Othman AE, Bach JP, Wiesmann M, Reich A (2018). Improvement of endovascular stroke treatment: a 24-hour neuroradiological on-site service is not enough. Biomed Res Int.

[18] Palli C, Fandler S, Doppelhofer K, Niederkorn K, Enzinger C, Vetta C (2017). Early dysphagia screening by trained nurses reduces pneumonia rate in stroke patients: a clinical intervention study. Stroke.

