# Supplementary material for: Presentation outside office hours does not negatively influence treatment times for reperfusion therapy for acute ischemic stroke
Source: J Neurol. 2020 Jul 31;268(1):133–9. doi: 10.1007/s00415-020-10106-z (PMC7815598; doi:10.1007/s00415-020-10106-z)
Supplement: Supplementary file 1 — Supplementary file1 (DOCX 21 kb) [file 415_2020_10106_MOESM1_ESM.docx]

Supplemental data

**Presentation outside office hours does not negatively influence treatment times for reperfusion therapy for acute ischemic stroke in the Greater Amsterdam Area**

A.E. Groot^1^, H. de Bruin^1^, T.T.M. Nguyen^2^, I.J. de Brouwer^1^, Manon Kappelhof^3^, F. de Beer^4^, M.C. Visser^5^, C.P. Zwetsloot^6^, Marieke de Graaf, P. Halkes^7^, J. de Kruijk^8^, W. van der Meulen^9^, T. van der Ree^10^, V.I.H. Kwa^11^, S. van Schaik^12^, L. Hani^13^, P.J. Nederkoorn^1^, C.B.L.M. Majoie^3^, Y.B.W.E.M. Roos^1^, J.M. Coutinho^1^.

| **Supplemental table 1. List of affiliations** | | |  |  |
| --- | --- | --- | --- | --- |
|  | **Hospital** | **Principal Investigator** | **Time period** | **Number of patients**  Included/total |
| 1. | Amsterdam University Medical Centers, location AMC | Prof. Dr. Y.B.W.E.M. Roos Dr. J. Coutinho | IVT: 2009-2015 EVT: 2014-2017 | 580/646 |
| 2. | Amsterdam University Medical Centers, location VUmc | Dr. M.C. Visser | 2009-2015 | 415/443 |
| 3. | MC Slotervaart | Dr. M.T. de Graaf | 2009-2015 | 176/187 |
| 4. | Onze Lieve Vrouwe Gasthuis, location Oost | Dr. V.I.H. Kwa | 2009-2015 | 318/329 |
| 5. | Onze Lieve Vrouwe Gasthuis, location West | Dr. S.M. van Schaik | 2009-2015 | 603/635 |
| 6. | Waterland Purmerend | C.P. Zwetsloot | 2009-2015 | 104/118 |
| 7. | Rode Kruis hospital Beverwijk | W.D.M. van der Meulen | 2009-2015 | 185/214 |
| 8. | Westfries Gasthuis Hoorn | T. van der Ree | 2009-2015 | 311/331 |
| 9. | Noordwestziekenhuisgroep Alkmaar | P.H.A. Halkes | 2009-2015 | 495/549 |
| 10. | Noordwestziekenhuisgroep Den Helder | L. Hani | 2012-2015 | 50/101 |
| 11. | Tergooi hospitals | J. de Kruijk | 2009-2015 | 344/352 |
| 12. | Spaarne Gasthuis, location Haarlem | F. de Beer | 2009-2015 | 492/575 |
| 13. | Spaarne Gasthuis, location Hoofddorp | F. de Beer | 2013-2015 | 155/171 |
| 14. | Flevoziekenhuis, Almere | Dr. M. Limburg | Only EVT patients | 15/22 |
| 15. | Boven’Ij | M. Janmaat | Only EVT patients | 1/3 |

| **Supplemental table 2. Outcomes for patients treated with endovascular treatment, stratified according to door of comprehensive stroke center** (n=6 excluded because of missing door time CSC) | | | | | |
| --- | --- | --- | --- | --- | --- |
|  | **Office hours n=146** | **Outside office hours**  **N=243** | **P-value** | **Unadjusted Beta/OR (95% CI)** | **Adjusted Beta*/OR****  **(95% CI)** |
| Median DGT (IQR) | 131 (96 – 176) | 127 (107 – 168) | 0.937 | -1.3 (-13.0 to 10.4) | -1.0 (-12.4 to 10.3) |
| Direct patients (n=129) | 89 (65 – 117) | 103 (73 – 122) | 0.187 | 6.7 (-11.1 to 24.5) | 5.6 (-12.7 to 23.8) |
| Transfer patients (n=260) | 158 (121 – 201) | 141 (118 – 180) | 0.169 | -8.6 (-21.6 to 4.4) | -6.0 (-19.4 to 7.5) |
| In-hospital mortality – n (%) | 23/146 (15.8) | 30/243 (12.3) | 0.343 | 0.75 (0.42 – 1.35) | 1.40 (0.62 – 3.15) |
| Mortality after 3 months – n (%) | 35/123 (28.5) | 65/212 (30.7) | 0.671 | 1.11 (0.68 – 1.81) | 1.50 (0.74 – 3.05) |
| mRS 0-2 after 3 months – n (%) | 57/135 (42.2) | 85/224 (37.9) | 0.422 | 0.84 (0.54 – 1.29) | 0.74 (0.41 – 1.32) |
| Direct patients (n=129) | 24/48 (50.0) | 31/70 (44.3) | 0.541 | 0.80 (0.38 – 1.66) | 0.70 (0.23 – 2.11) |
| Transfer patients (n=260) | 33/87 (37.9) | 54/154 (35.1) | 0.656 | 0.88 (0.51 – 1.52) | 0.71 (0.34 – 1.50) |
| Symptomatic ICH – n (%) | 13/117 (11.1) | 20/191 (10.5) | 0.860 | 0.94 (0.45 – 1.96) | 0.97 (0.42 – 2.22) |
| Pneumonia – n (%) | 16/146 (11.0) | 26/243 (10.7) | 0.936 | 0.97 (0.50 – 1.88) | 1.96 (0.79 – 4.86) |
| Urinary tract infection – n (%) | 4/146 (2.7) | 10/243 (4.1) | 0.481 | 1.52 (0.47 – 4.95) | 2.09 (0.56 – 7.78) |
| ICU admission – n (%) | 38/146 (26.0) | 39/243 (16.0) | 0.017 | 0.54 (0.33 – 0.90) | 0.80 (0.41 – 1.56) |
| Abbreviations: DGT, door-to-groin time; IQR, interquartile range; SD, standard deviation; ICH, intracranial hemorrhage; ICU, intensive care unit.  *Adjusted for age, sex, prior ischemic stroke/TIA, use of antithrombotic therapy, pre-stroke mRS, baseline NIHSS score, systolic blood pressure, onset-to-needle time. **Adjusted for age, sex, IVT treatment, prior ischemic stroke/TIA, use of antithrombotic therapy, pre-stroke mRS, baseline NIHSS, onset-to-needle time. | | | | | |
